# Supplementary material for: Molecular Identification of Asian Hornet Vespa velutina nigrithorax Prey from Larval Gut Contents: A Promising Method to Study the Diet of an Invasive Pest
Source: Animals (Basel). 2023 Feb 1;13(3):511. doi: 10.3390/ani13030511 (PMC9913685; doi:10.3390/ani13030511)
Supplement: Supplementary file 1 [file animals-13-00511-s001.zip › animals-2125298-supplementary/Table S1.pdf]

Table S1: List of common taxa found among nests analysed and the number of larval samples containing hits for each taxon.

Group	Taxon	Jersey	Tetbury	Alderney	Gosport	Wool.
**Honey bee**	*Apis mellifera*	9/9	3/10	-	3/3	10/10
**Asian hornet**	*Vespa velutina*	1/9	1/10	-	-	-
**Wasp**	*Vespula spp.*	2/9	9/10	6/6	3/3	6/10
**Blow fly**	*Calliphora spp.*	9/9	2/10	6/6	-	4/10
**Blow fly**	*Lucilia spp.*	6/9	1/10	4/6	2/3	-
**Blow fly**	*Pollenia spp.*	6/9	6/10	-	1/3	10/10
**Blow fly**	*Stomorhina lunata*	1/9	-	-	-	-
**Hover fly**	*Eristalis spp.*	3/9	-	3/6	-	4/10
**Hover fly**	*Eumerus spp.*	2/9	-	-	-	-
**Hover fly**	*Scaeva spp.*	-	-	2/6	-	-
**Hover fly**	*Myathropa florea*	3/9	-	-	-	-
**Hover fly**	*Sericomyia spp.*	-	-	1/6	-	-
**Hover fly**	*Syrphus spp.*	-	-	3/6	-	-
**Hover fly**	*Volucella pellucens*	1/9	-	-	-	-
**House fly**	*Helina spp.*	1/9	4/10	-	1/3	-
**House fly**	*Musca spp.*	1/9	-	-	-	-
**House fly**	*Phaonia spp.*	-	1/10	-	-	-
**House fly**	*Polietes spp.*	-	-	-	-	2/10
**Fruit fly**	*Drosophila suzukii*	-	-	-	1/3	-
**Noon fly**	*Mesembrina meridiana*	-	1/10	-	-	-
**Dung fly**	*Scathophaga spp.*	2/9	-	-	-	4/10
**Flesh fly**	*Sarcophaga spp.*	4/9	-	-	1/3	1/10
**Common Fly**	*Dryomyza anilis*	-	6/10	-	-	-
**Soldier fly**	*Sargus bipunctatus*	-	1/10	-	-	-
**Tachinid fly**	*Tachinidae spp.*	5/9	-	1/6	-	-
**Spider**	*Araneus diadematus*	-	8/10	-	1/3	10/10
**Spider**	*Metellina segmentata*	-	2/10	-	-	-
**Spider**	*Zygiella spp.*	-	-	1/6	1/3	-
**Grasshopper**	*Acrididae spp.*	-	-	-	-	1/10
**Mosquito**	*Ochlerotatus detritus*	-	-	-	1/3	-
**Moth**	*Phlogophora meticulosa*	-	-	1/6	-	-
**Fungus**	*Monodus spp.*	-	-	-	3/3	-
**Hedgehog**	*Erinaceus europaeus*	-	1/10	-	-	-
**Woodlouse**	*Oniscus asellus*	-	1/10	-	-	-
**Bacteria**	*Uncultured bacterium*	1/9	6/10	0/6	3/3	1/10
